# A Novel Metric System to Quantify Antibiotic Consumption in Paediatric Population: A Hospital Based, Biphasic Pilot Study

**DOI:** 10.15190/d.2020.16

**Published:** 2020-12-10

**Authors:** Shah Newaz Ahmed, Ratinder Jhaj, Ritendra Patidar, Mahendra Dangi, Shikha Malik, Balakrishnan Sadasivam, Shubham Atal

**Affiliations:** ^1^Department of Pharmacology, All India Institute of Medical Sciences, Bhopal, Madhya Pradesh, India; ^2^Department of Paediatrics, All India Institute of Medical Sciences, Bhopal, Madhya Pradesh, India

**Keywords:** Drug utilisation, pediatric, antibiotic consumption, metric system, defined daily dose.

## Abstract

BACKGROUND: The Anatomical Therapeutic Chemical Classification / Defined Daily Dose (ATC/DDD) system recommended by World Health Organization is accepted worldwide as the standard method of quantification of drug consumption. However, owing to individual variation in body weight, the ATC/DDD system cannot be used for comparison across paediatric population. OBJECTIVE: This study aimed to develop a novel metric system for standard quantification of antibiotic consumption in paediatric population. METHOD: The standard unit of drug quantification in adult population is DDD/100 patient days (PD). We conceived a new unit of DDD/1000 kg-days (KD) where KD is the product of the body weight and length of hospital stay of an individual patient. We simulated the quantification and comparison of drugs in a computer model of five virtual paediatric hospitals (H1 to H5, n=100, 200, 100, 100, 100 respectively). We re-applied the metric system on two, real world, hospital-based, time cohorts (TC) (TC18, n=38 and TC19, n=47) of 2 weeks each, in two consecutive years. RESULTS: The body weights (mean±SD) in H1-H5 were 5.7±3.0, 5.7±2.8, 25.3±8.5, 20.6±11.7 and 19.8±11.4 kg, respectively. The antibiotic consumption in terms of DDD/100 PD and DDD/1000 KD in the five hospitals was 1.26, 1.20, 5.52, 4.41 and 2.00, and 2.24, 2.14, 2.22, 2.17 and 1.06 respectively. In TC18 and TC19, the mean body weight, DDD/100 PD and DDD/1000 KD were 12.24±13.17, 30.93, 20.34 and 19.51±12.28, 11.99, 6.23, respectively. CONCLUSION: DDD/1000 kg-days is a potential standard unit for drug quantification in paediatric population independent of weight distribution and size of the study sample. The universal application and comparison across diverse samples can generate useful information for resource allocation, anti-microbial stewardship, disease burden and drug use, and can help in taking policy decisions to improve healthcare delivery in the paediatric population.

## INTRODUCTION

The Anatomical Therapeutic Chemical Classification / Defined Daily Dose (ATC/DDD) system for quantification and comparison of drug consumption, recommended by World Health Organization (WHO) is considered as the international standard^1^. While the ATC seeks to standardize the classification of drugs, the DDD is defined as the assumed average maintenance dose per day for a drug used for its main indication in adults. The DDD provides a fixed unit of measurement independent of price and formulation^2^. The purpose of the ATC/DDD system is to serve as a tool for drug utilization monitoring and research in order to improve quality of drug use^3^. In adult population, the commonly used unit of drug consumption for quantification and comparison is DDD/100 Patients Days (PD). The denominator in the unit is PD. One patient staying in the hospital for one day is equivalent to one patient day. The use of DDD/100 PD ensures comparability between studied adult cohorts. However, there is a major hurdle in use of units based on PD in paediatric population, owing to variation in body weight of the individual patients^4^. With the objective of developing a standard measurement unit in this special population, we conceived a novel denominator for DDD calculation. Instead of PD, we calculated the sum total of weight-time products of the individual patients in units of kg-days and quantified the antibiotic consumption in terms of DDD/1000 kg-days. Before enacting the novel method of quantification in real world scenario, we simulated a model using random computer-generated data for virtual hospital-based cohorts. The favorable results of the model led us to conduct an investigational pilot study to judge the feasibility and utility of the novel metric system in paediatric drug audit.

## METHODS

### Quantification of Patient Burden

#### Unit in Adults

The standard unit of patient burden in hospitals is PD. In general, the number of PD is calculated as one less than the number of days from the day of admission to the day of discharge. The most common denominator for indicating antibiotic consumption in terms of DDD in adult population is 100 PD^5^.

#### Novel Unit in Children

Unlike adult population, 100 PD is not a suitable denominator for indicating antibiotic consumption in the paediatric population, as stated above, due to variation in body weight of the individual patients. We propose a novel unit for quantifying patient burden in paediatrics using a multiplication product of patient’s body weight and length of hospital stay (weight-time product). The summation of weight-time product of all the patients will be representative of patient burden. We propose to use 1000 kg-days as the denominator for indicating the antibiotic consumption in DDDs in paediatric population.

### Standard Unit of Drug Consumption

*Drug usage in DDD units* =

(number of doses per day **x** number of days of therapy **x** amount of drug per dose)** /** DDD

*DDD/100 patient days* =

(sum-total of drug usage in DDD units of all the patients in the cohort **/** number of patient days in the cohort referred in the numerator) **x** 100

*Days of therapy (DOT)* =

number of days for which one drug in the recommended dose is administered to a patient

### Novel unit of Drug Consumption in Children

*DDD/1000 kg-days* =

(sum-total of drug usage in DDD units of all the patients in the cohort **/** sum-total of weight-time products of all the patients in the cohort in units of kg-days) **x** 1000

### Simulation of the Novel Metric System

#### Principle

In the standard unit of drug consumption, DDD/100 PD, the drug consumed in units of DDD is expressed with respect to 100 PD. This denominator ensures comparability in adult population where the standard daily doses are administered per person. In the paediatric population, where doses are administered per kg body weight for individual patients, the denominator of 100 PD may lead to misleading interpretation. This is especially true when we compare drug consumption in two paediatric samples with dissimilar weight distribution. In order to eliminate this discrepancy, a novel unit, namely DDD/1000 kg-days, wherein the denominator is the sum-total of the products of body weight and length of stay of the individual patients, is proposed. The unit is deemed to reflect drug consumption independent of size and distribution of a sample and therefore, can be an indispensable tool for drug utilisation monitoring in paediatric population.

#### Data Generation

Data were generated randomly for five virtual hospital cohorts (numbered H1 to H5) representing a sample of 100, 200, 100, 100 and 100 paediatric patients in the weight bands of 1-10, 1-10, 10-40, 1-40 and 1-40 kg respectively. Each virtual patient received a randomly assigned antibiotic (coded between 1 to 10) for a randomly assigned number of days (range 1 to 10). In hospital 5, only 50% of the patients received antibiotics. The DDD of the antibiotics received pre-defined values between 0.1 to 12 grams. The paediatric dose was derived as the quotient of the DDD divided by adult reference body weight (assumed to be 60 kg). All data for the simulation work was generated in Microsoft Excel, which permits random data generation. The syntax for random data generation for a required range and frequency, and assignment of values to variables is described in our previous work^6^.

#### Simulation Output

The number of patients in Hospital 1 (H1), Hospital 2 (H2), Hospital 3 (H3), Hospital 4 (H4) and Hospital 5 (H5) was 100, 200, 100, 100 and 100 respectively. The mean body weight (mean ± SD) of the patients in the five hospital cohorts was 5.7±3.0, 5.7±2.8, 25.3±8.5, 20.6±11.7 and 19.8±11.4 kg respectively. The patient burden in terms of patient days and kg-days and the antibiotic consumption in terms of DOT, DDD, DDD/100 PD and DDD/1000 kg-days is shown in Table 1.

**Table 1:** Patient burden and antibiotic consumption in the five virtual hospitals

	Hospital 1	Hospital 2	Hospital 3	Hospital 4	Hospital 5
*Number of patients*	100	200	100	100	100
*Weight Band in Kg*	01-10	01-10	10-40	01-40	01-40
*Total Patient Days*	754	1594	763	778	800
*Total Kg-days*	4228	8912	18976	15801	15126
*DOT*	754	1594	763	778	405
*DDD*	9.47	19.05	42.12	34.33	16.02
*DDD/100 PD*	1.26	1.20	5.52	4.41	2.00
*DDD/1000 Kg-days*	2.24	2.14	2.22	2.17	1.06

### Data Collection

The study was carried out in the Paediatric Inpatient Department of a 800 bedded tertiary care centre in Central India. Data was collected in two phases (15 days each) 9 months apart. The data was collected from prescription records only. It included patient particulars, diagnosis and antibiotics used, including dose, frequency and route of administration. Data was anonymised and stored in password protected electronic devices. The data from prescription records in each phase constituted a time-cohort (TC). The data in the first and second phase were collected in May 2018 (TC18) and March 2019 (TC19) respectively. The study did not involve participation of human subjects. Drugs were coded as per ATC classification system with the help of information available at *https://www.whocc.no/*, the official site of WHO Collaborating Centre for Drug Statistics Methodology, Oslo, Norway. Prior permission from the Institutional Human Ethics Committee was obtained for the conduct of the study.

### Data Analysis

Data from the two phases were analysed separately and compared. The number of patients, gender distribution, mean age, mean body weight and mean length of hospital stay, in the two TCs was computed. The focal analysis consisted of a direct comparison between the two TCs with respect to the patient burden in terms of patient days and kg-days, and the antibiotic consumption in terms of DDD, DDD/100 PD, DOT and DDD/1000 kg-days.

## RESULTS

The number of patients in TC18 and TC19 was 38 (boys/girls=21/17) and 47 (boys/girls=27/20) respectively. The mean (mean ± SD) body weight (in kg) in the two cohorts was 12.24±13.17 and 19.51±12.28 respectively. The median length of hospital stay was 9 and 8 days respectively. The patient burden and total antibiotic consumption in the two TCs are shown in Table 2. The consumption of individual antibiotics is shown in Table 3.

**Table 2:** Patient burden and antibiotic consumption

Time-Cohort	Body Weight in kg (Mean±SD)	Patient Burden - Patient days	Patient Burden - Kg-days	Antibiotic Consumption - DOT	Antibiotic Consumption - DDD	Antibiotic Consumption - DDD/100PD	Antibiotic Consumption - DDD/1000 kg-days
TC18 (n=38)	12.24±13.17	308	4320	312	87.85	30.93	20.34
TC19 (n=47)	19.51±12.28	395	7596	100	47.32	11.99	6.23

**Table 3:** Consumption of individual antibiotics in TC18 and TC19 cohorts, in the four different units Defined Daily Dose (DDD); days of therapy (DOT); patient days (PD); kg-days (KD); time-cohort (TC); not prescribed (XX); *1 unit dose (UD) of cotrimoxazole = sulfamethoxazole 0.4 g/trimethoprim 80 mg.

No.	Antibiotic	TC18	TC18	TC18	TC18	TC19	TC19	TC19	TC19
		DOT	DDD	DDD / 100PD	DDD / 1000 KD	DOT	DDD	DDD / 100PD	DDD / 1000 KD
01	Doxycycline J01AA02 DDD-0.1 g	6	12	4.23	2.78	XX	XX	XX	XX
02	Chloramphenicol J01BA01 DDD-3 g	8	0.27	0.09	0.06	XX	XX	XX	XX
03	Amoxicillin -clavulanic acid J01CR02 DDD-3 g	18	2.01	0.71	0.47	16	5.32	1.35	0.70
04	Piperacillin-tazobactam J01CR05 DDD-14 g	22	0.68	0.24	0.16	XX	XX	XX	XX
05	Meropenem J01DH02 DDD-2 g	12	4.50	1.58	1.04	XX	XX	XX	XX
06	Imipenem J01DH51 DDD-2 g	4	0.48	0.17	0.11	XX	XX	XX	XX
07	Cefotaxime J01DD01 DDD-4 g	12	1.26	0.44	0.29	XX	XX	XX	XX
08	Ceftriaxone J01DD04 DDD-2 g	85	36.07	12.70	8.35	44	29.86	7.56	3.93
09	Cefixime J01DD08 DDD-0.4 g	32	6.55	2.31	1.52	9	8.00	2.03	1.05
10	Cotrimoxazole J01EE01 DDD-4 UD *	5	0.33	0.12	0.08	12	0.54	0.14	0.07
11	Azithromycin J01FA10 DDD-0.5 g	9	2.40	0.85	0.56	XX	XX	XX	XX
12	Clindamycin J01FF01 DDD-1.8 g	3	0.17	0.06	0.04	XX	XX	XX	XX
13	Amikacin J01GB06 DDD-1 g	21	3.42	1.20	0.79	14	2.10	0.53	0.28
14	Vancomycin J01XA01- 2 g	34	4.94	1.74	1.14	XX	XX	XX	XX
15	Amphotericin B J02 AA01 DDD-35 mg	4	1.14	0.40	0.26	XX	XX	XX	XX
16	Fluconazole J02AC01 DDD-0.2 g	23	10.85	3.82	2.51	XX	XX	XX	XX
17	Metronidazole P01AB01 DDD-2 g	14	0.78	0.27	0.18	5	1.50	0.38	0.20
	**TOTAL**	**312**	**87.85**	**30.93**	**20.34**	**100**	**47.32**	**11.99**	**6.23**

## DISCUSSION

In our computer-generated data, we advertently created the virtual cohorts with varying distribution of body weight, number of patients, Patient Days and Days of Therapy to mimic real world conditions. In the simulation output, despite similar consumption in terms of DOT, cohorts with body weights in higher range showed greater consumption in terms of DDD/100 PD (DOT/DDD 100PD for H1, H3 and H4- 754/1.26, 763/5.52 and 778/4.41 respectively). H3 (weight range: 10-40 kg) and H2 (weight range 1-10 kg) showed the maximum and minimum consumption in terms of DDD/100 PD respectively. Clearly, DDD/100 PD is not the correct representation of antibiotic usage in these paediatric cohorts with different body weight ranges. Using our novel metric system, the antibiotic consumption for H1, H2, H3, H4 and H5 was 2.24, 2.14, 2.22, 2.17 and 1.06 DDD/1000 kg-days respectively. This parameter ensures comparability between the cohorts and is independent of body weight and sample size. Importantly, H5 (weight range: 1-40 kg) showed greater consumption than H1 and H2 (weight range: 1-10 kg) in terms of DDD/ 100 PD (2.00 versus 1.26 and 1.20) but lesser consumption in terms of DDD/1000 kg-days (1.06 versus 2.24 and 2.14). It is to be noted that only 50 out of 100 patients in H5 received antibiotic in contrast to H1 where all the patients received antibiotics. In H1 and H2, despite different number of patients and days of therapy, antibiotic consumption was similar in both the units of DDD/100 PD and DDD/1000 kg-days. This was the case because of the same weight distribution of patients in the two cohorts. Being a unit independent of body mass of individual patients, DDD/1000 kg-days can serve as a standard unit of quantification and comparison of drug consumption in paediatric drug audit.

We conducted a biphasic pilot study to test the effectiveness of the novel metric system in real world conditions. Quantification and comparison of antibiotic consumption in the two phases (TC18 and TC19) ratified that the new unit can be a suitable tool in paediatric drug audit. The patient burden in TC18 (n=38) was less than TC19 (n=47), both in terms of patient-days and kg-days. However, the antibiotic consumption was greater in TC18 in all the three units of quantification. The consumption in terms of DOT in TC18 and TC19 was 312 and 100 respectively. DOT reflects the pattern of antibiotic use but provides incomplete information on the quantity of the drug used^7^. In units of DDD/100 PD, the consumption in TC18 and TC19 was 30.93 and 11.99 respectively. Though DDD/100 PD seeks to quantify the amount of drug used, it cannot be used to compare the consumption between TC18 and TC19 because the two time cohorts differ significantly in their mean body weights (12.24±13.17 versus 19.51±12.28, P<0.05). Finally, the consumption in the two-time cohorts in terms of DDD/1000 kg-days was 20.34 and 6.23 respectively. This unit with denominator of kg-days is independent of body weight and sample size of the study cohorts and can be used for comparisons and for deriving meaningful conclusions.

The development of a standard unit that can be universally and uniformly used in all paediatric age groups has been a long-desired goal in drug utilisation studies. In neonatal population, a metric system of neonatal DDD based on an assumed neonatal weight of 2 kg was conceived^8^. However, the system remained limited to the referred neonatal sub-population and failed to ensure uniformity in different paediatric age groups. In the absence of a standard unit, most of the research work conducted in paediatric audit have fallibly utilised DDD/100 PD for drug quantification. Elena et al evaluated antibiotic consumption in paediatric population (in terms of DDD/PD) in a 8-year survey (2004-2011) and found that antibiotic consumption increased non-significantly (P=0.224) during the period. The authors lamented the lack of a standard unit for drug quantification in paediatric population and emphatically stated that “DDD is intended for adult population and that paediatric age includes children who need different antibiotic doses depending on their body weight”^9^. Problems in paediatric drug audit are augmented during comparative studies and systematic reviews. Most of the time, drug consumption across paediatric populations are rendered non-comparable owing to variable weight distribution of the samples. Systematic reviews which requires pooling of data to arrive at a single absolute measure of the outcome of interest becomes difficult. The authors of a systematic review synthesized data from 79 drug audit studies involving paediatric patients and found that the most frequently used units of measurement were DDD/PD and exposed patients/patients. In attempts to correlate the antibiotic consumption with antibiotic resistance (obtained as r=0.83), they hopefully reiterated that a standard measure of antimicrobial use in paediatric population will be available in the future and will better predict antimicrobial resistance in surveillance studies^10^. In a first of its kind, multicentric study, Porta et al. computed antibiotic consumption in four standardised weight bands (neonatal, <10, 10-25, >25 kg) and used the data for inter-centric comparison^4^. Taking a cue from this pioneering study, in our previously published work, we had developed a novel metric system based on quantifying drug consumption in terms of DDD/100 PD in discrete weight categories. Though a very robust unit for comparison and quantification, it involves segregating the sample into multiple discrete weight based sub-samples, and then quantifying and comparing drug consumption across similar sub-samples of other audits^6^. In the present work, we have developed yet another standard unit, which is deemed to be effective in smaller samples and unlike our previous metric system, is free from exhaustive computational burden.

## CONCLUSION

DDD/1000 kg-days is a novel unit for drug quantification in paediatric population independent of weight distribution and size of the study sample. We feel it has the potential to be adopted as a standard unit for drug utilisation studies in the paediatric population. The universal application and comparison across diverse samples can generate useful information for resource allocation, anti-microbial stewardship, disease burden and drug use, and can help in taking policy decisions to improve healthcare delivery in this special population.
